# Supplementary material for: Microinterfaces in biopolymer-based bicontinuous hydrogels guide rapid 3D cell migration
Source: Nat Commun. 2024 Mar 29;15:2766. doi: 10.1038/s41467-024-46774-y (PMC10980809; doi:10.1038/s41467-024-46774-y)
Supplement: Supplementary file 1 — Supplementary Information [file 41467_2024_46774_MOESM1_ESM.pdf]

Supplementary Information

Microinterfaces in biopolymer-based bicontinuous hydrogels guide rapid 3D cell migration

Karen L. Xu[1,2,3,4], Nikolas Di Caprio[1,2], Hooman Fallahi[5], Mohammad Dehghany[2,6], Matthew D. Davidson[2,7,8], Lorielle Laforest[3,4], Brian C. H. Cheung[9], Yuqi Zhang[1,3,4], Mingming Wu[9], Vivek Shenoy[2,6], Lin Han[5], Robert L. Mauck[1,2,3,4], Jason A. Burdick[1,2,7,8]

[1]Department of Bioengineering, University of Pennsylvania, Philadelphia, PA, 19104, USA
[2]Center for Engineering Mechanobiology, University of Pennsylvania, Philadelphia, PA, 19104, USA
[3]McKay Orthopaedic Research Laboratory, Department of Orthopaedic Surgery, Perelman School of Medicine, University of Pennsylvania, Philadelphia, PA, 19104, USA
[4]Translational Musculoskeletal Research Center, Corporal Michael J. Crescenz VA Medical Center, Philadelphia, PA, 19104, USA
[5]School of Biomedical Engineering, Science and Health Systems, Drexel University, Philadelphia, 19104, PA, USA
[6]Department of Materials Science and Engineering, University of Pennsylvania, Philadelphia, PA 19104, USA.
[7]BioFrontiers Institute, University of Colorado Boulder, Boulder, CO, 80303, USA
[8]Department of Chemical and Biological Engineering, University of Colorado Boulder, Boulder, CO, 80303, USA
[9]Department of Biological and Environmental Engineering, Cornell University, Ithaca, NY, 14850, USA

Corresponding Authors: jason.burdick@colorado.edu and lemauck@pennmedicine.upenn.edu

Supplementary Information

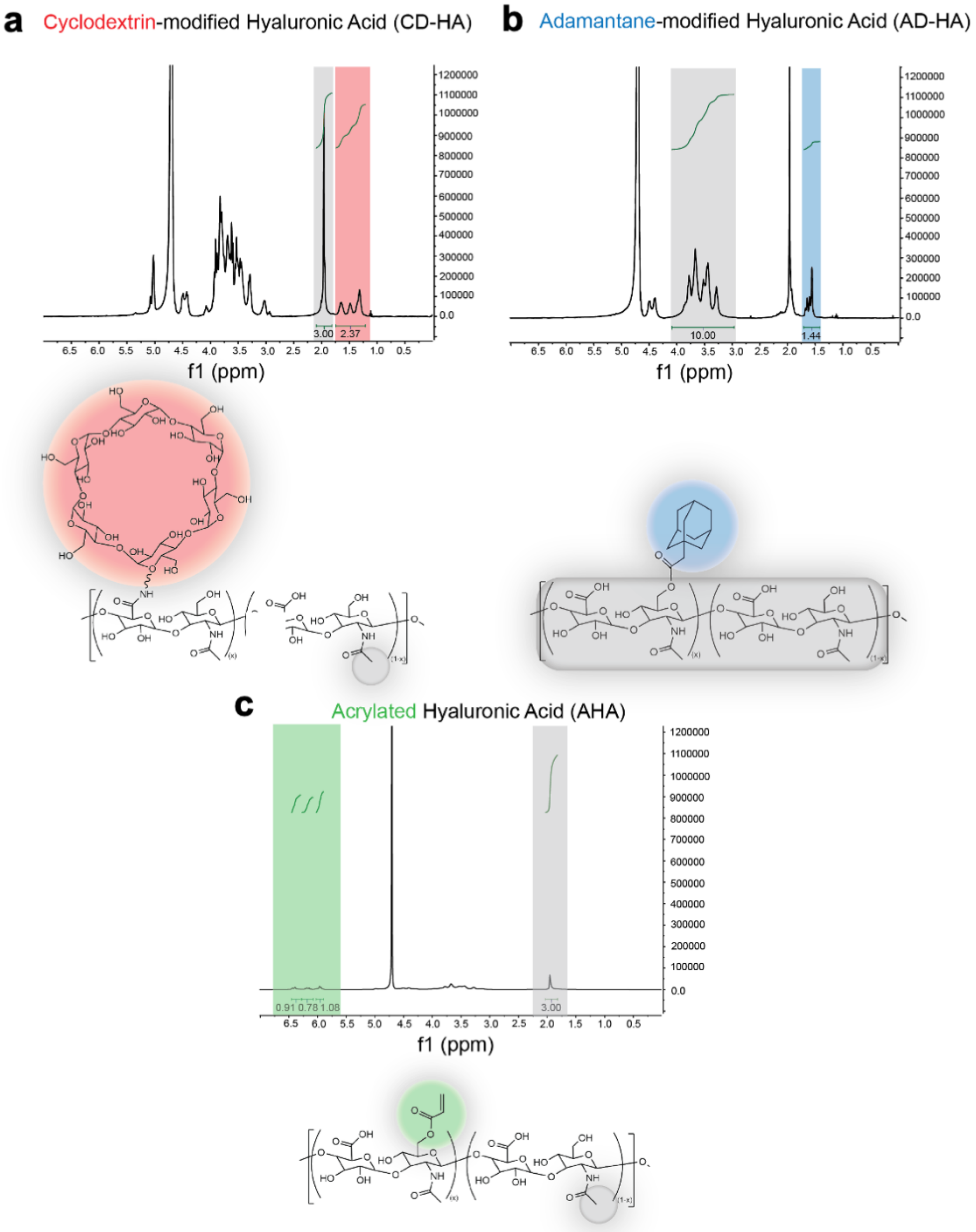

Supplementary Fig. 1: ^{1}H NMR spectra of synthesized CD-HA, AD-HA, and AHA. a, Chemical structure and ^{1}H-NMR spectrum of cyclodextrin-modified hyaluronic acid (CD-HA). Modification of CD was determined by the integration of the hexane linkers (Red, 12H, δ: 1.2-1.75 ppm) when normalized to methyl group (Grey, 3H, 1.7-2.0 ppm). **b,** Chemical structure and ^{1}H-NMR spectrum of adamantane-modified hyaluronic acid (AD-HA). Modification of AD was determined by the integration of the ethyl multiplet (Blue, 12H, δ: 1.4-1.7 ppm) when normalized to the HA backbone (Grey, 10H, δ: 2.9-4.0 ppm). **c,** Chemical structure and ^{1}H-NMR spectrum of acrylated hyaluronic acid (AHA). Modification of AHA was determined by the integration of the acrylate peaks (Green, 3H, δ: 5.9-6.1 ppm, 6.1-6.3 ppm, and 6.3-6.5 ppm) when normalized to the methyl group on HA (Grey, 3H, δ: 1.7-2.0 ppm).

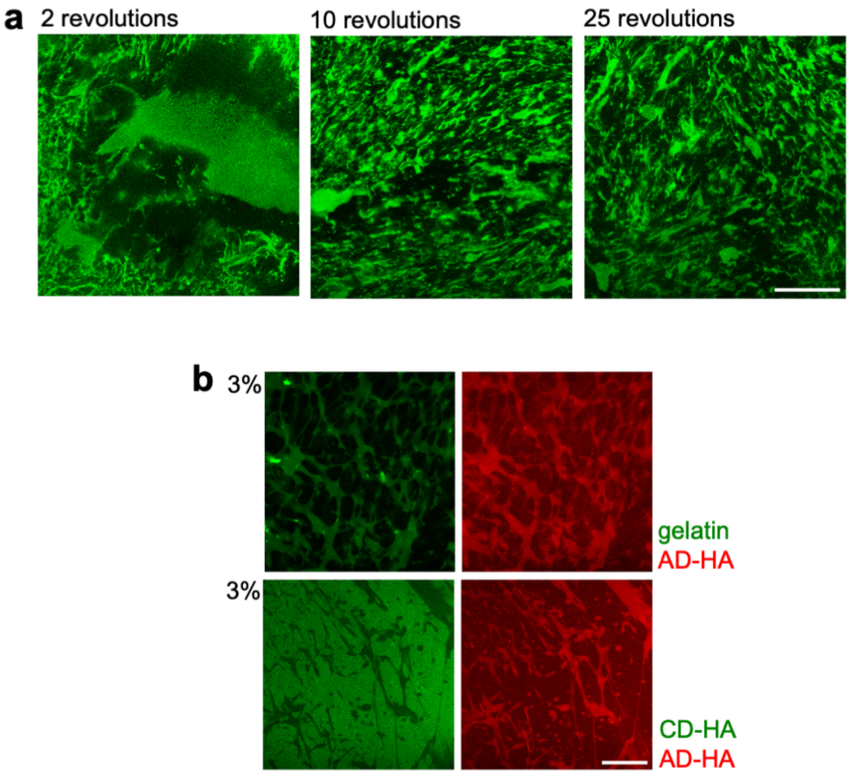

Supplementary Fig. 2: Bicontinuous hydrogel structure relies on sufficient mixing of two immiscible polymer solutions. a, Representative single Z-sections of bicontinuous hydrogels with variations in the extent of mixing (i.e., number of revolutions). Scale bar = 50 µm. **b**, Representative single z-sections of a 3% bicontinuous hydrogel with selective fluorescent labeling of different hydrogel constituents, in which either gelatin (green) and AD-HA (red) are labeled (top) or CD-HA (green) and AD-HA (red) are labeled (bottom). Scale bar = 50 µm.

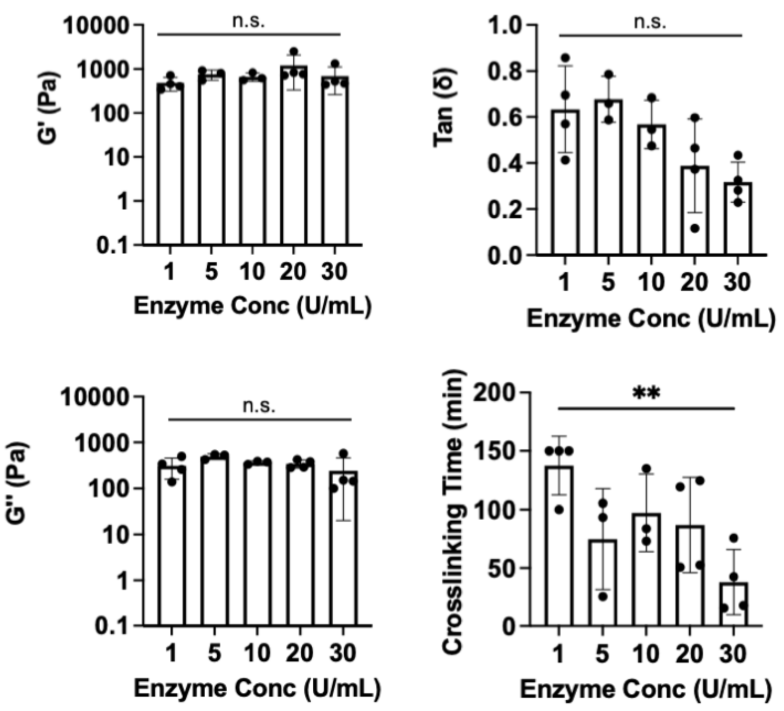

Supplementary Fig. 3: Rheologic mechanical properties of bicontinuous hydrogels with varying enzyme concentrations. Rheological measurements (1 Hz, 1% strain) of hydrogels (5wt% gelatin, 3wt% GH) with varying enzymatic crosslinker (1,5,10,20,30 U/mL) including storage modulus (top, left panel), loss modulus (bottom, left panel), tan (delta)(top, right panel), and time required for G' to reach 99% of its final modulus (bottom, right panel). n= 3 (5,10 U/mL) or 4 (1, 20, 30 U/mL) hydrogels per condition. Top Left, Top Right, Bottom Left panel: n.s. indicates no statistical significance. Bottom Right panel: 1 vs. 30 ***p*=0.0087; one-way ANOVA with Tukey post hoc. Data are mean ± s.d. Source data provided as a source data file.

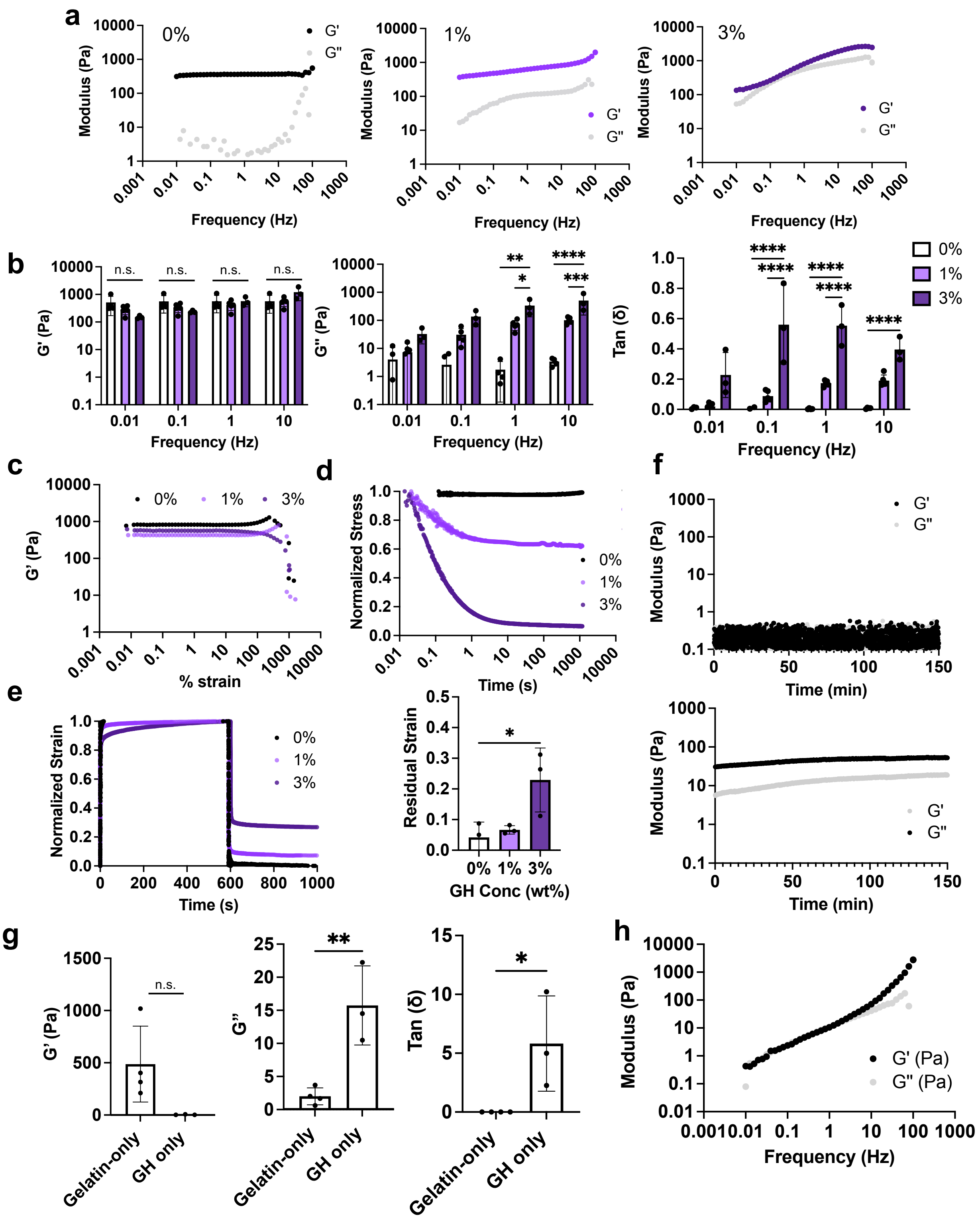

Supplementary Fig. 4: Bicontinuous hydrogels are viscoplastic and stress-relaxing. a, Representative frequency sweeps (0.01–100 Hz, 1% strain). **b,** Rheological measurements extrapolated from frequency sweeps across different frequencies (0.01, 0.1, 1, 10 Hz, 1% strain)

of storage modulus (left panel), loss modulus (middle panel), and tan (delta) (right panel). n= 3 (3%), 4 (0%) or 5 (1%) hydrogels per condition. Left panel: n.s. indicates no statistical significance. Middle panel: 1 Hz: 0% vs. 3% $^{**}p=0.043$; 1 Hz: 1% vs. 3% $^{*}p\leq0.0335$; 10 Hz: 0% vs. 3% $^{****}p\leq0.0001$; 10 Hz: 1% vs. 3% $^{***}p=0.0001$. Right panel: 0.1 Hz: 0% vs. 3%, 1% vs. 3% $^{****}p\leq0.0001$; 1 Hz: 0% vs. 3%, 1% vs. 3% $^{****}p\leq0.0001$; 10 Hz: 0% vs. 3%: $^{****}p\leq0.0001$; two-way ANOVA with Tukey post hoc. **c,** Representative strain sweeps (1 Hz, 0.001 to 1000% strain). **d,e,** Representative stress relaxation (**d**, 10% strain) and creep-recovery (**e**-left panel, 100 Pa) studies and quantification of residual strain from creep-recovery studies (**e**-right panel). n= 3 hydrogels per condition. $^{*}p=0.0331$, one-way ANOVA with Tukey post hoc. **f,** Representative gelation kinetics (1 Hz, 1% strain) of 5 wt% gelatin without enzymatic crosslinker or GH (top panel) and of 5wt% gelatin with GH but without enzymatic crosslinker (bottom panel). **g,** Quantification of G', G'', and tan (delta) for gelatin (data of 0% group from **Fig. 1e**) and GH hydrogel. n= 4 hydrogels per condition. Left panel: n.s. indicates no statistical significance. Middle Panel: $^{**}p=0.005$; Right panel: $^{*}p=0.031$; two-tailed unpaired students t-test. **h,** Representative frequency sweep of 3 wt% GH hydrogel (0.01–100 Hz, 1% strain). Data are mean ± s.d. Source data for (**a-h**) provided as a source data file.

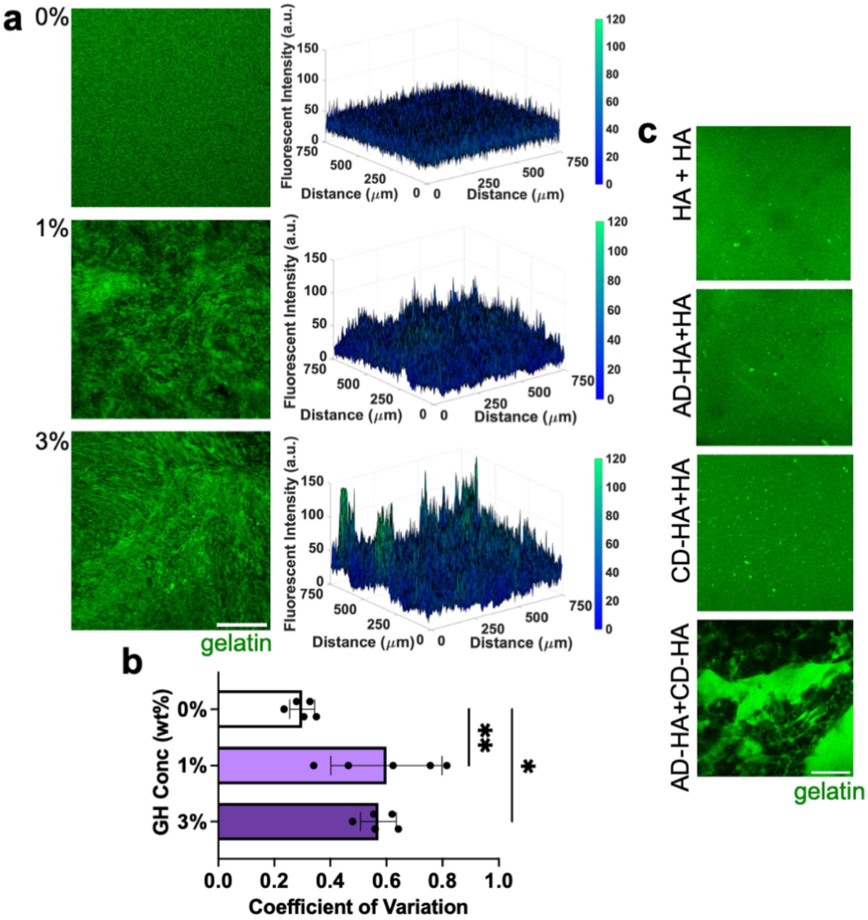

Supplementary Fig. 5: Bicontinuous hydrogel structure evolves with increasing GH concentration and relies on both GH components. a, Representative single Z-sections of bicontinuous hydrogels (left panel) separated into GR (green) and GP (unlabeled) domains and their corresponding fluorescent intensity profiles (right panel). Scale bar = 200 µm. **b,** Quantification of variation in structural properties of GR domains (green). n= 5 regions across 3 distinct gels per condition. 0% vs. 1% ***p*=0.0059; 0% vs. 3% **p*=0.0114; one-way ANOVA with Tukey post hoc. **c,** Representative single Z sections of bicontinuous hydrogel structures based on presence of each GH component. Scale bar = 100 µm. Data are mean ± s.d. Source data for (**b**) provided as a source data file.

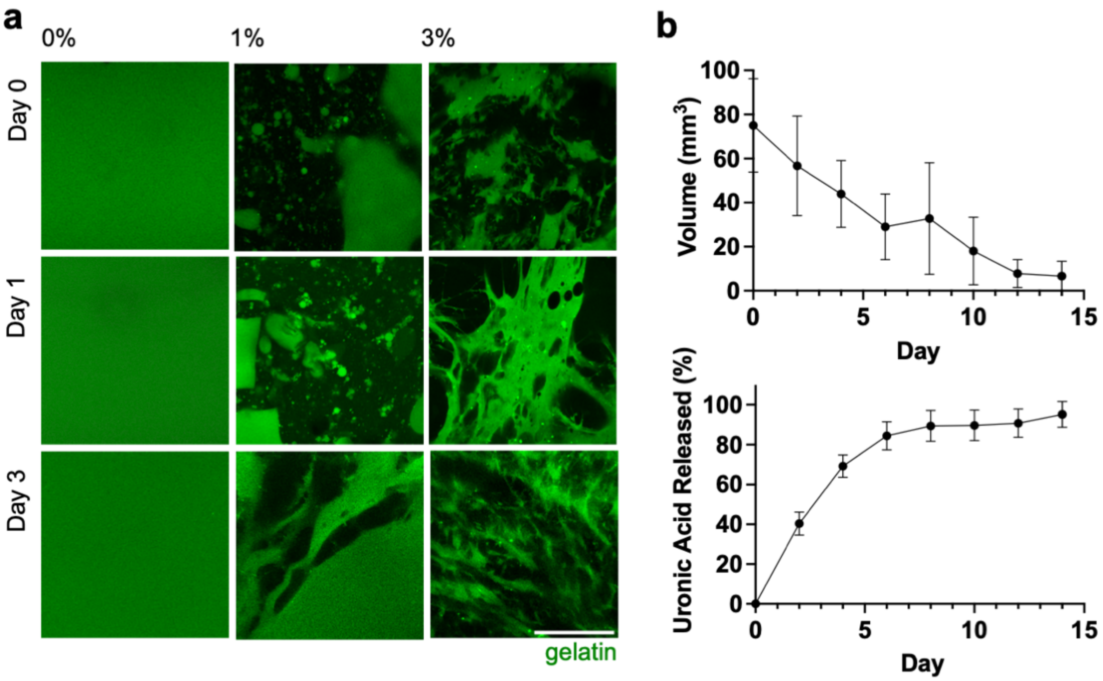

Supplementary Fig. 6: Bicontinuous hydrogels remain structurally and chemically stable over several days. a, Bicontinuous hydrogel structure where GR (green) and GP (unlabeled) remain distinct over 3 days. Scale bar = 100 µm. **b,** Hydrogel volume (top panel) and cumulative uronic acid released (bottom panel) of a 3% bicontinuous hydrogel in PBS over time. Data are mean ± s.d. Source data for (**b**) provided as a source data file.

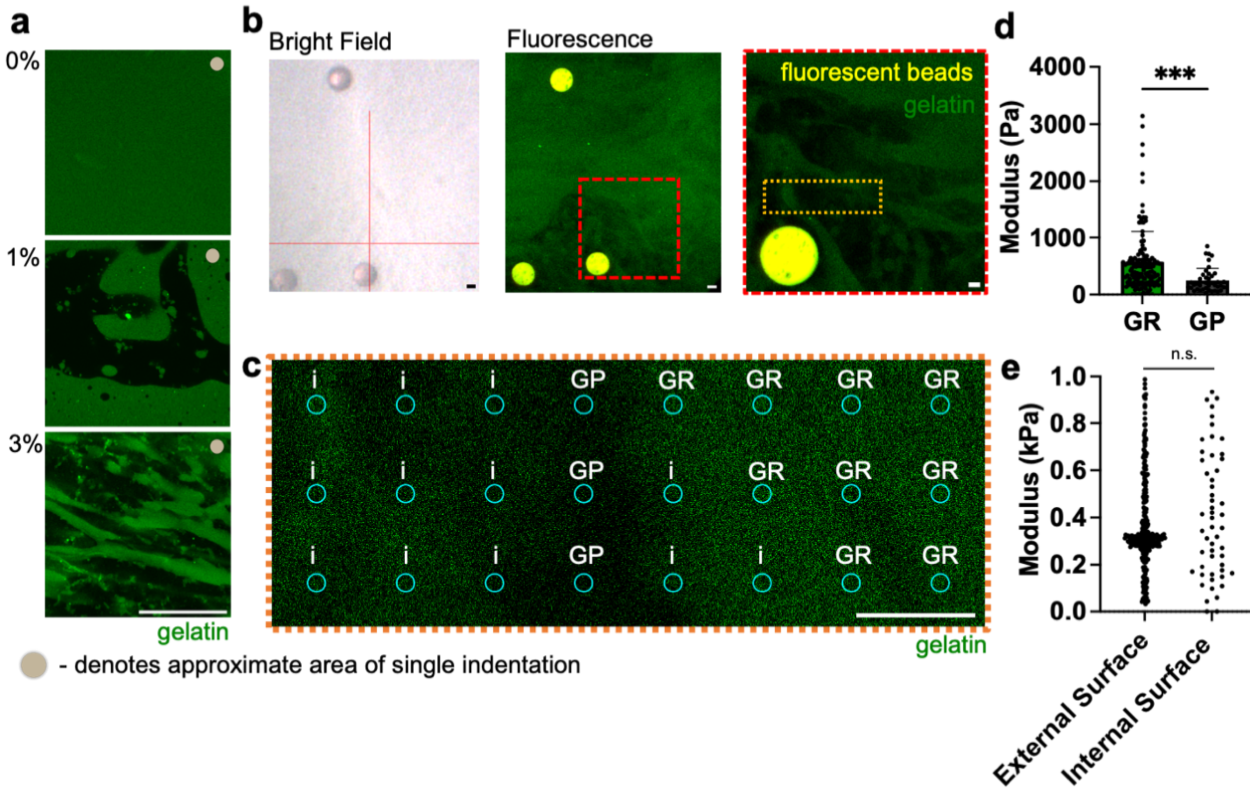

Supplementary Fig. 7: Methodology for quantifying differential mechanical properties of GR and GP domains. a, Representative fluorescent images (GR:green) denoting approximate area of indentation. Scale bar = 50 μm. **b,c,** Workflow of quantification of mechanical properties in different fluorescent regions. Bright field images were taken during AFM nanoindentation (**b-left panel**, red cross represents cantilever tip; fiduciary bead-red). Fluorescent images (**b-middle panel**) of hydrogels (GR domains: green, GP domains: unlabeled) were then correlated to bright field images based on fiduciary beads (yellow). Red dashed line denotes zoom-in area corresponding to zoom-in representative fluorescent image **(b-right panel).** Orange dashed denotes further zoom-in area corresponding to analyzed grid **(c),** where indentation location is approximately denoted by cyan circle. i – inconclusive; GR – gelatin rich; GP - gelatin-poor. Scale bar = 20 μm. **d,** Elastic modulus of individual indentations based on differential fluorescent areas. n≥ 41 points across 5 hydrogels. ****p*=0.0003; two-tailed unpaired student's t-test. **e,** Comparison of AFM-nanoindentation modulus between external (top surface) and internal (gel sectioned in half and exposed surface examined) surfaces of a 1% GH bicontinuous hydrogel. n≥ 96 indentations from 1 (internal surface) or 3 (external surface) hydrogels. n.s. indicates no statistical significance; two-tailed unpaired student's t-test. Data are mean ± s.d. Source data for (**d,e**) provided as a source data file.

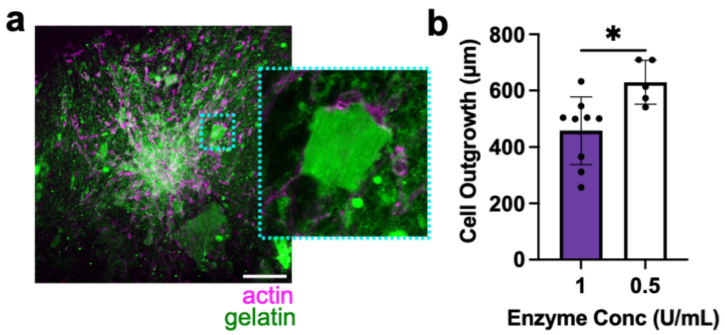

Supplementary Fig. 8: Lowering enzymatic crosslinker does not affect interface-based guidance of cell migration. a, Representative maximum projection of cells (actin:magenta) migrating from spheroid along interfaces between GR (green) and GP domains. Inset (dotted blue border) is a single Z-section highlighting cells along GR domain. Scale bar = 200 µm. **b,** Quantification of cell outgrowth of 3% bicontinuous hydrogel with 1 U/mL (data from Fig. 3d) or 0.5 U/mL of transglutaminase. n= 5 (0.5) or 9 (1) spheroids per condition from 2 biologically independent experiments. **p*=0.0147, two-tailed unpaired student's t-test. Data are mean ± s.d. Source data for (**b**) provided as a source data file.

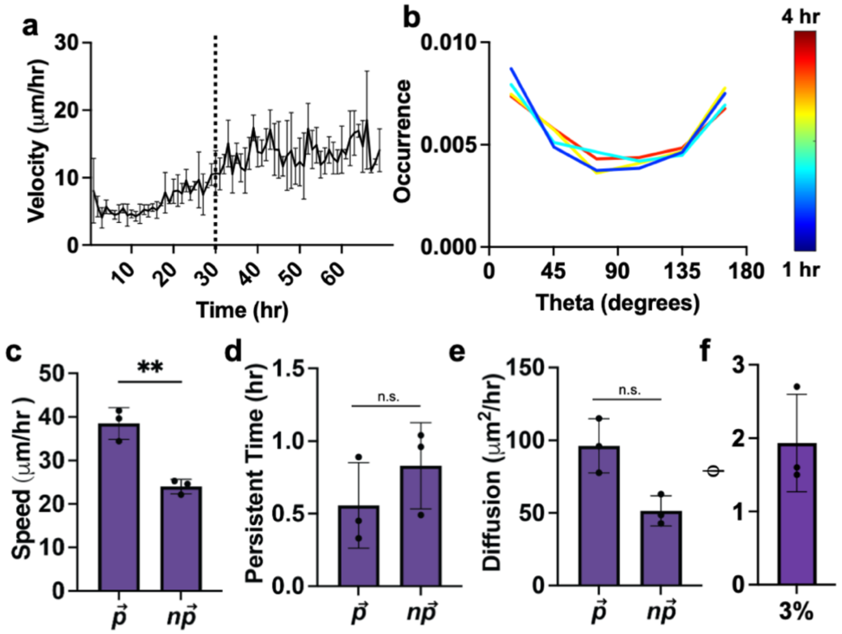

Supplementary Fig. 9: Characterization of cell migration directionality in 3% bicontinuous hydrogel. a, Population-averaged cell speed is time invariant once cells leave spheroid (Time ~30 hours, denoted with dashed line), a requirement for implementation of the APRW model. **b,** Representative distributions of angular displacements of cell outgrowth after specified time lag (denoted with heat map) of 3% GH hydrogel within one spheroid. **c-e,** Migration speed (**c**), persistence **(d),** and diffusion **(e)** along primary migration axis ($\vec{p}$) and nonprimary migration axis ($n\vec{p}$) of cells averaged per spheroid in 3% GH hydrogels. n= 3 spheroids across 1 biologically independent experiment. c: ***p*=0.0014. d,e: n.s. indicates no statistical significance; two-tailed paired student's t-test. **f,** Anisotropic index ϕ calculated from ratio of diffusion along $\vec{p}$ and $n\vec{p}$**.** n= 3 spheroids across 1 biologically independent experiment. n.s. indicates no statistical significance, two-tailed paired student's t-test. Data are mean ± s.d. Source data for (**a-f**) provided as a source data file.

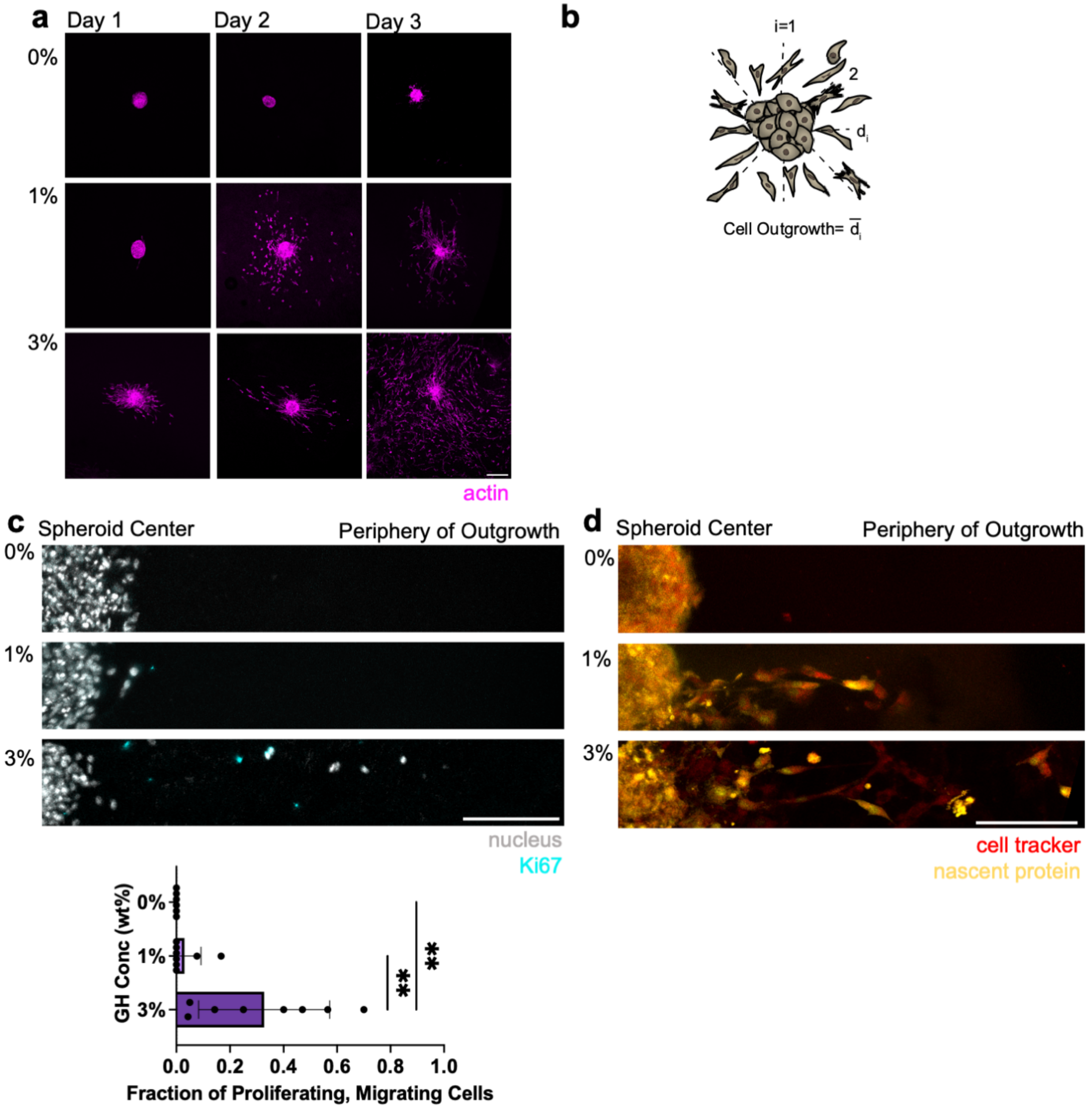

Supplementary Fig. 10: MFC cell migration, proliferation and nascent protein deposition. a, Representative images of cell outgrowth via actin (magenta) over time. Scale bar = 200 µm. **b,** Schematic demonstrating quantification of spheroid outgrowth. **c,** Representative images of Ki67 stain (cyan) with nuclei mask (white) over 3 days (**c-top panel,** Scale bar = 100 µm) and corresponding quantification (**c-bottom panel**). n= 6 (0%) or 8 (1,3%) spheroids per condition from 2 biologically independent experiments. 0% vs. 3% ***p*=0.0023; 1% vs. 3% ***p*=0.0027; one-way ANOVA with Tukey post hoc. **d**, Representative images of nascent protein deposition (yellow) with cell tracker (red) over 3 days. Scale bar = 100 µm. Data are mean ± s.d. Source data for (**c**) provided as a source data file.

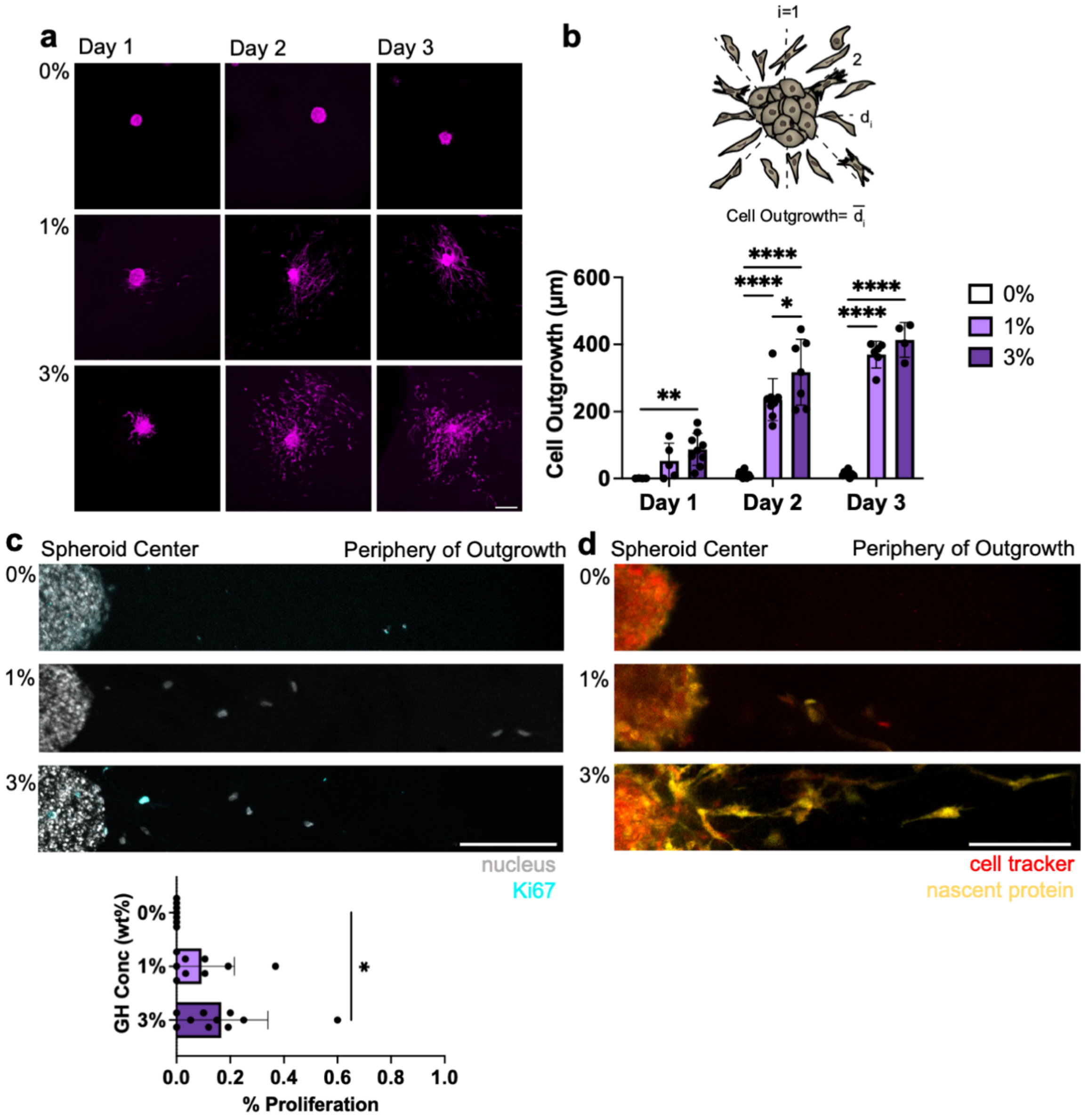

Supplementary Fig. 11: MSC cell migration, proliferation and nascent protein deposition. **a,** Representative images of cell outgrowth via actin (magenta) over 3 days. Scale bar = 200 μm. **b,** Schematic demonstrating quantification of spheroid outgrowth (top panel), and quantification of MSC cell outgrowth over time (bottom panel). n= 4 (Day 3:3%), 5 (Day 1:1%), 6 (Day 3:1), 7 (Day 2:3%), 8 (Day 2:1%), 9 (Day 1:0%,3%), 12 (Day 3:0%), or 14 (Day 2:0%) spheroids per condition across 2 biologically independent experiments. Day 1: 0% vs. 3% ***p*=0.0037; Day 2: 0% vs. 1%, 0% vs. 3% *****p*≤0.0001; Day 2: 1% vs. 3% **p*=0.0227; Day 3: 0% vs. 1%, 0% vs. 3% *****p*≤0.0001; two-way ANOVA with Tukey post hoc. **c,** Representative images of Ki67 stain (cyan) with nuclei mask (white) over 3 days (**c-top panel,** Scale bar = 100 μm) and corresponding quantification (**c-bottom panel**). n= 7-10 spheroids per condition across 2 biologically independent experiments. **p*=.0418; one-way ANOVA with Tukey post hoc. **d**, Representative images of nascent protein deposition (yellow) with cell tracker (red) over 3 days. Scale bar = 100 μm. Data are mean ± s.d. Source data for (**b,c**) provided as a source data file.

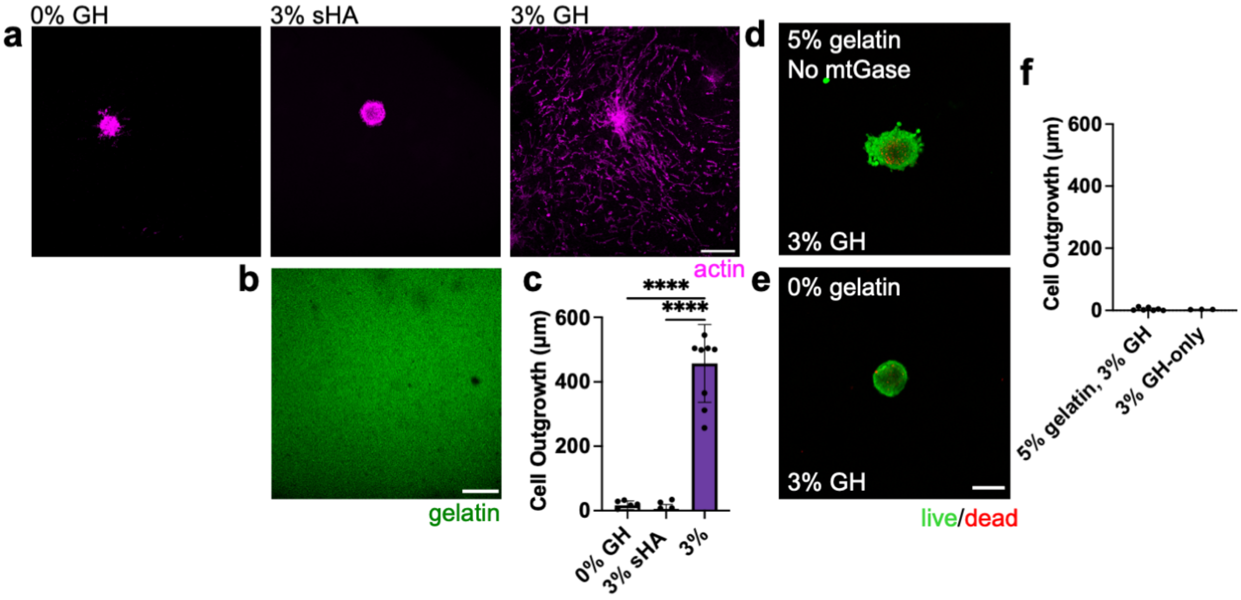

Supplementary Fig. 12: The presence of HA alone does not account for increased migration. a, Representative images of cell (actin: magenta) outgrowth at day 3 in 0 wt% GH, 3 wt% soluble HA (sHA), or 3% bicontinuous hydrogel (all conditions with 5 wt% gelatin and 1 U/mL transglutaminase, 3% from Fig. 3c). Scale Bar = 200 µm. **b,** Gelatin (green) distribution in hydrogels with soluble HA. Scale Bar = 200 µm. **c,** Quantification of cell outgrowth in sHA group compared to 3% bicontinuous hydrogel (data from Fig. 3d). n= 6 (0%), 9 (3%) or 10 (1%) spheroids per condition from 2 biologically independent experiments. ****$p \leq 0.0001$, one-way ANOVA with Tukey post hoc. **d,e,** Live (green)-Dead (red) of MFC spheroids in 3 wt% GH and 5 wt% (**d**) or 0 wt% gelatin (**e**). Scale Bar = 200 µm. **f,** Quantification of cell outgrowth based on Live/Dead stain in 3% GH, and 5% gelatin, 3% GH-only hydrogels (all conditions without transglutaminase). Data are mean ± s.d. Source data for (**c**,**f**) provided as a source data file.

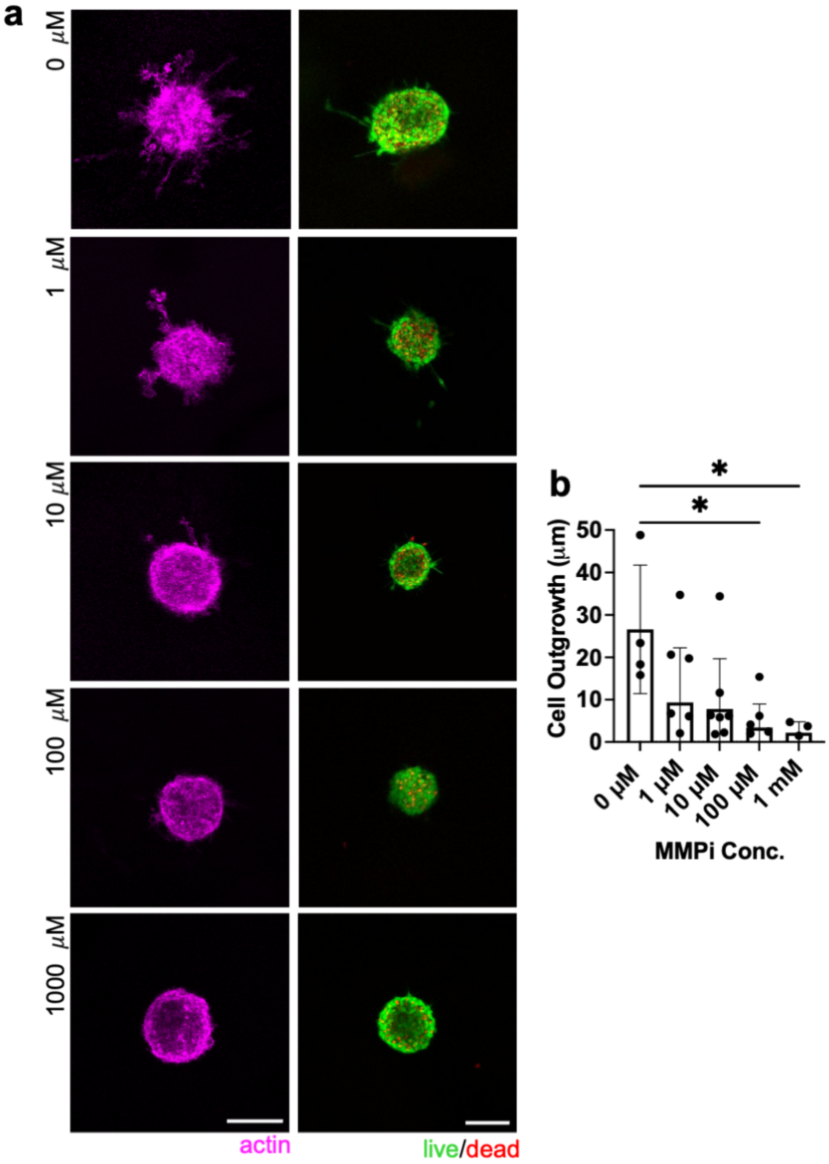

Supplementary Fig. 13: Cell outgrowth via protease-dependent mechanisms can be tuned through MMP inhibitor concentration. a, Representative images of actin (magenta) in 0% GH hydrogel after 3 days with varying Marimastat concentrations (left panels) and Live (green)-Dead (red) of MFC spheroids in corresponding inhibitor groups (right panels). Scale bar = 100 μm **b,** Quantification of cell outgrowth into 0 wt% GH hydrogels. n= 4 (0,1 mM), 8 (10,100 μM) or 9 (1 μM) spheroids per condition from 1 biologically independent experiment. 0 vs. 100 μM $*p=0.0123$; 0 vs. 1 mM $*p=0.0264$; one-way ANOVA with Tukey post hoc. Data are mean ± s.d. Source data for (**b**) provided as a source data file.

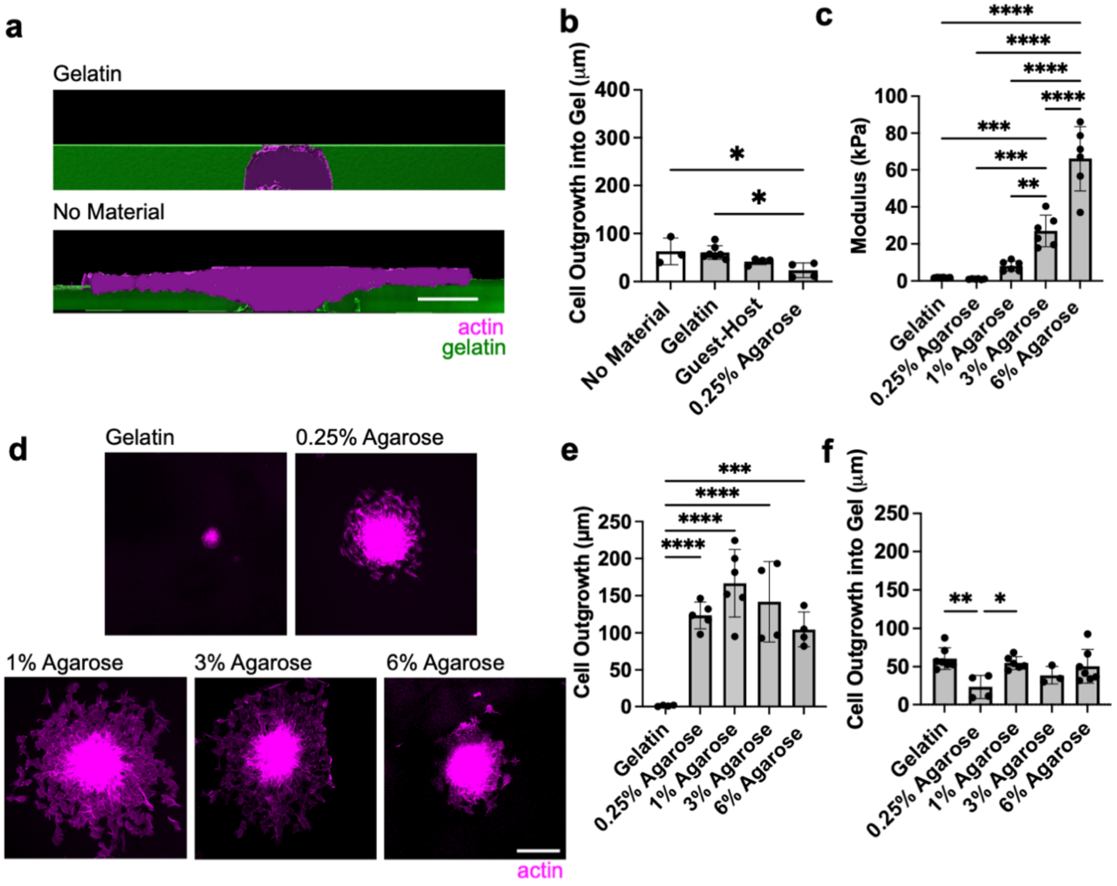

Supplementary Fig. 14: Cells infiltrate along engineered agarose interfaces despite increasing differential mechanical properties across the interface. a, Side view of 3D reconstructions of spheroids (magenta) spreading on gelatin surface (green) after 1 day. Scale bar = 80 μm**. b,** Perpendicular outgrowth from spheroids corresponding to Fig. 5a-d. n= 3 (No Material, 4 (GH, 0.25% Agarose), or 7 (Gelatin) from 2 biologically independent experiments. Note that quantification is from spheroid center, which is why there are variations depending on the material. No Material vs. 0.25% Agarose *p=0.0266; Gelatin vs. 0.25% Agarose *p=0.0109; one-way ANOVA with Tukey post hoc. **c,** Compression modulus of gelatin (5wt%, 1 U/mL enzymatic crosslinker), and varying wt% of agarose. n= 6 hydrogels per condition. Gelatin vs. 3% Agarose ***p≤0.0004; 0.25% vs. 3% Agarose ***p≤0.0002; 1% vs. 3% Agarose **p=0.0081; Gelatin vs. 6% Agarose, 0.25% vs. 6% Agarose, 1% vs. 6% Agarose, 3% vs. 6% Agarose ****p≤0.0001; one-way ANOVA with Tukey post hoc. **d, e,** Representative top-down images of spheroids (magenta) migrating along interface created with gelatin and top layer after 1 day (**d**, Scale bar = 200 μm) and corresponding quantification (**e**). n= 4 (3,6% Agarose), 5 (0.25% Agarose), or 6 (1% Agarose, Gelatin) spheroids per condition across 2-3 biologically independent experiments. Gelatin vs. 0.25% Agarose, Gelatin vs. 1% Agarose, Gelatin vs. 3% Agarose ****p≤0.0001; Gelatin vs. 6% Agarose ***p=0.0008; one-way ANOVA with Tukey post hoc. **f,** Perpendicular outgrowth from spheroids. n= 3 (3% Agarose), 4 (0.25% Agarose), 6 (1% Agarose) or 7 (Gelatin, 6% Agarose) spheroids per condition across 2-3 biologically independent experiments. Gelatin vs. 0.25% Agarose **p=0.0087; 0.25% vs. 1% Agarose ***p=0.0389, one-way ANOVA with Tukey post hoc. Data are mean ± s.d. Source data for (**b,c,e,f**) provided as a source data file.

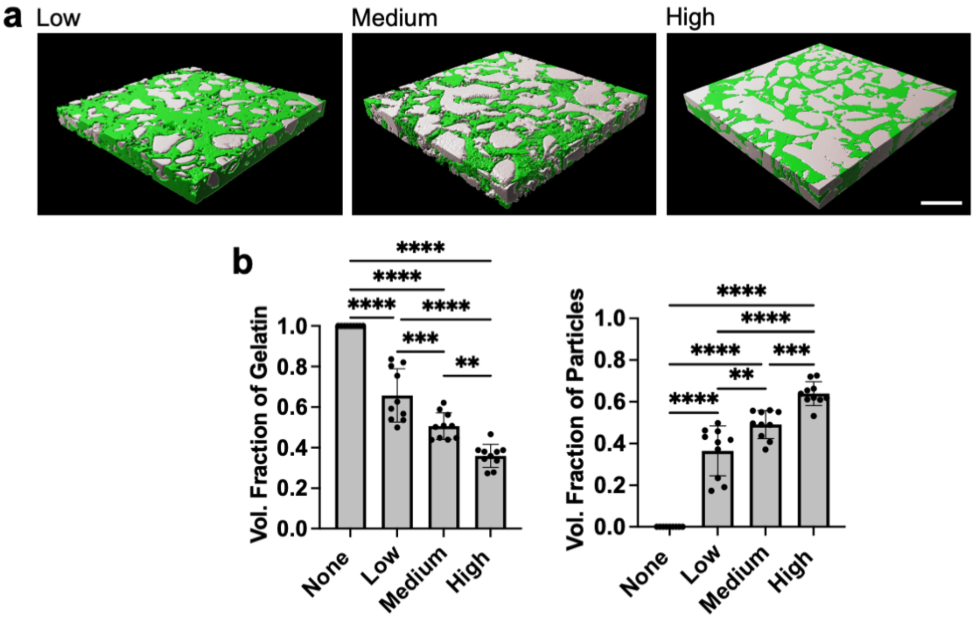

Supplementary Fig. 15: Additional structural characterization of gelatin-agarose particle composite hydrogels. a, Representative 3D reconstructions of hydrogels with varying densification (Low, Medium, High) of agarose particles within gelatin continuous phase (gelatin: green; agarose particles: gray). Scale bar = 100 μm. **b,** Fraction of total volume occupied by gelatin (left panel) and agarose particles (right panel) within hydrogels. n= 9 (None) or 10 (Low, Medium, High) regions across 3 hydrogels per condition. Right panel: None vs. Low, None vs. Medium, None vs. High, Low vs. High ****$p \leq 0.0001$; Low vs. Medium ***$p=0.0009$. Left Panel: None vs. Low, None vs. Medium, None vs. High, Low vs. High ****$p \leq 0.0001$; Low vs. Medium **$p=0.0035$; Medium vs. High ***$p=0.0005$; one-way ANOVA with Tukey post hoc. Data are mean ± s.d. Source data for (**b**) provided as a source data file.

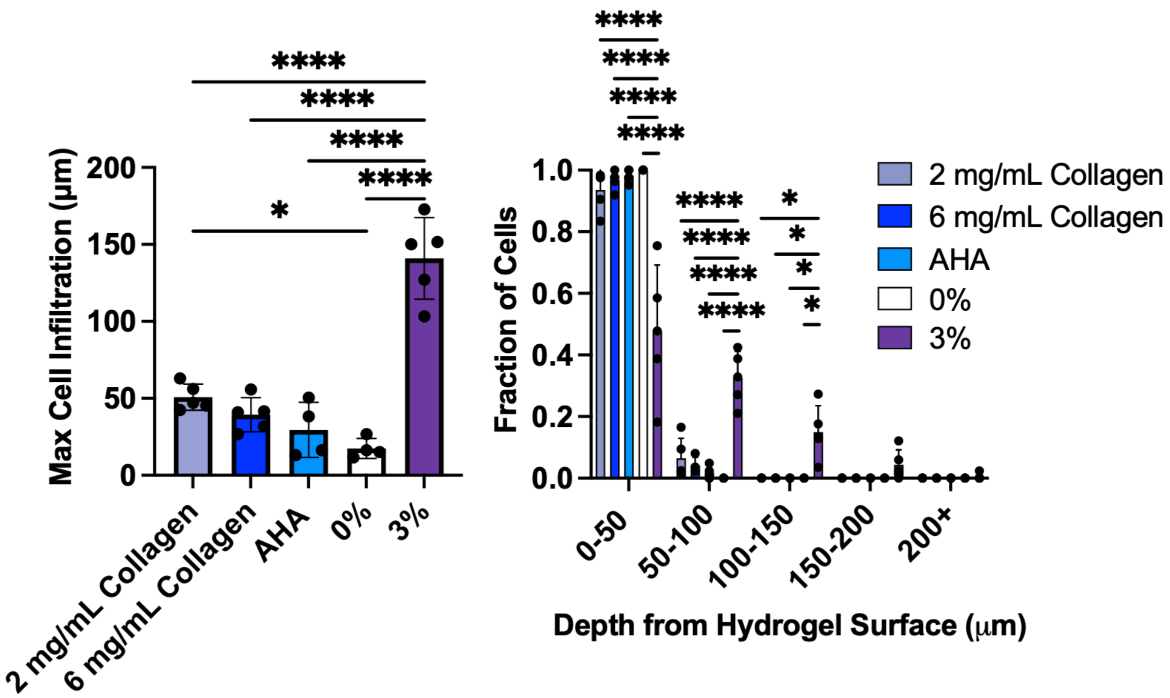

Supplementary Fig. 16: Cell infiltration from explants into uniform hydrogels. a, Quantification (0%, 3% groups from Fig. 6c) of maximum infiltration (left) and extent of cell infiltration through hydrogel depth (right). n= 4 (AHA, 0%) or 5 (2 mg/mL, 6 mg/mL, 3%) explants per condition from one biologically independent experiment. Left panel: 2 mg/mL collagen vs. 0% **p*=0.045; 2 mg/mL Collagen vs. 3%, 6 mg/mL Collagen vs. 3%, AHA vs. 3%, 1% vs. 3% *****p*≤0.0001; one-way ANOVA with Tukey post hoc. Right panel: 0-50: 2 mg/mL Collagen vs. 3%, 6 mg/mL Collagen vs. 3%, AHA vs. 3%, 0% vs. 3% *****p*≤0.0001; 50-100: 2 mg/mL Collagen vs. 3%, 6 mg/mL Collagen vs. 3%, AHA vs. 3%, 0% vs. 3% *****p*≤0.0001; 100-150: 2 mg/mL Collagen vs. 3%, 6 mg/mL Collagen vs. 3% **p*=0.0185; AHA vs. 3%, 0% vs. 3% **p*=0.0386; two-way ANOVA with Tukey post hoc (right). Data are mean ± s.d. Source data provided as a source data file.

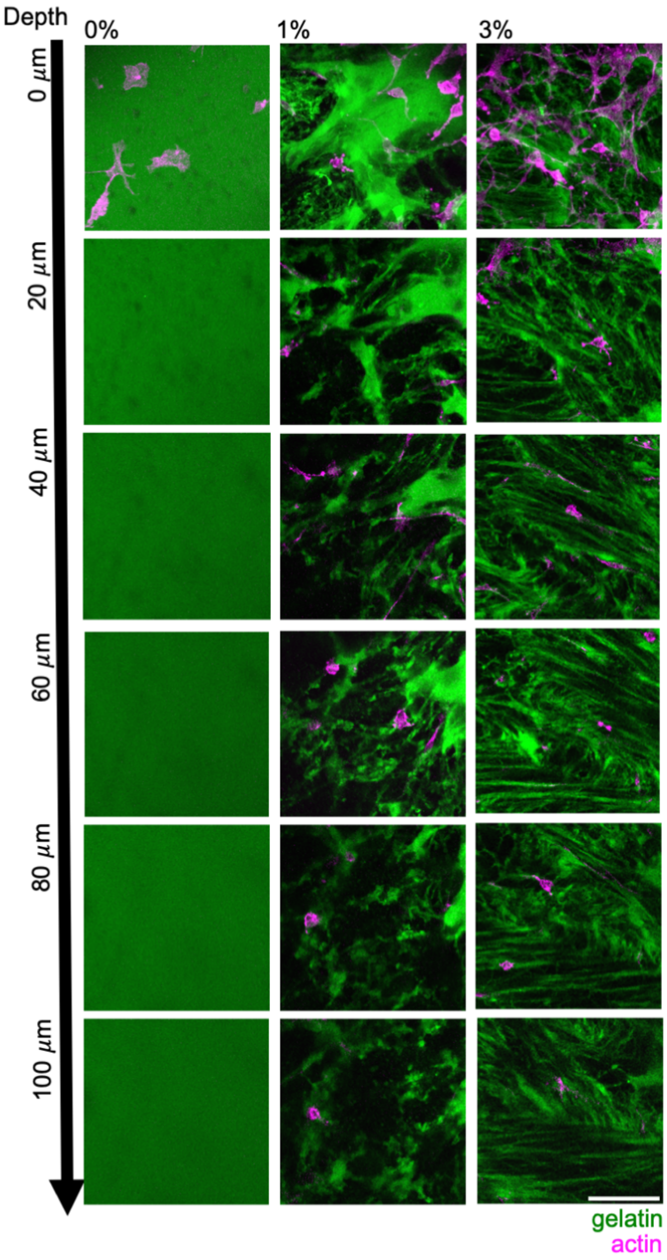

Supplementary Fig. 17: Cell infiltration from meniscus explants is influenced by microinterfaces. Single z-slices at different depths into the hydrogel (GR: green, GP: unlabeled) of ex vivo studies with single cell (magenta) infiltration. Scale Bar = 100 μm.

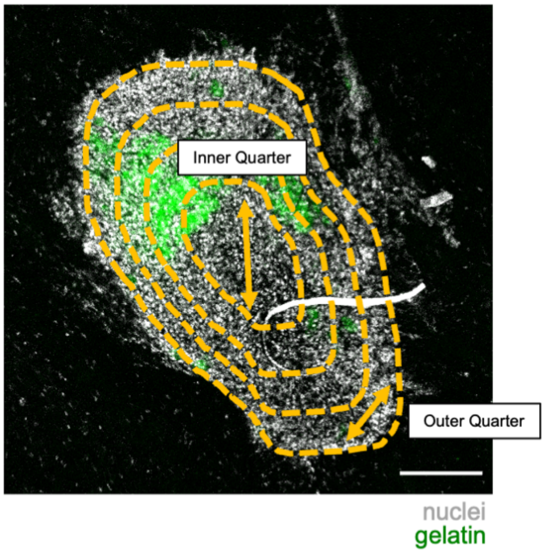

Supplementary Fig. 18: Methodology for quantifying cell density within quartiles into in vivo defect space. Representative in vivo maximum Z-projection of cells (nuclei: white, hydrogel: green) denoting binned areas for quantification (orange dashed line). Scale bar = 200 μm

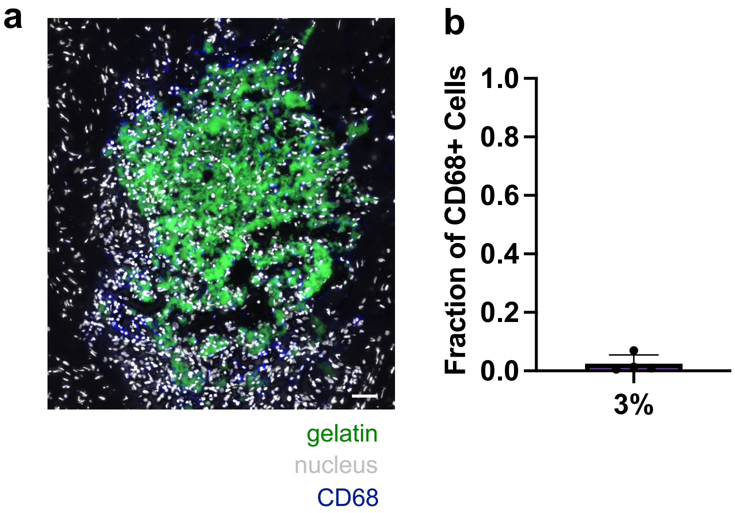

Supplementary Fig. 19: Cell infiltration from non-meniscus cells in vivo is minimal. a, Representative fluorescent image of remaining hydrogel (gelatin:green), CD68+ cells (blue) and cell nuclei (gray) and **b**, corresponding quantification of percentage of infiltrating cells that are CD68+ 14 days after implantation**.** n= 4 explants from 3 rats. Scale Bar = 100 µm. Source data for (**b**) provided as a source data file.

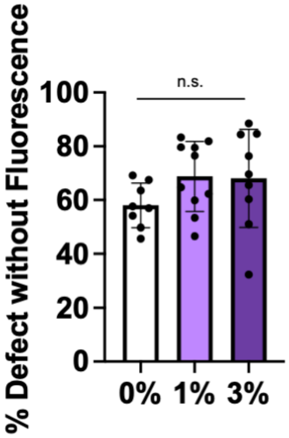

Supplementary Fig. 20: Gelatin fluorescence within defects. Quantification of fluorescence loss in vivo 14 days after subcutaneous implantation. n= 8 (0%), 9 (3%) or 10 (1%) explants per condition across 8 rats. n.s. indicates no statistical significance, one-way ANOVA with Tukey post hoc. Data are mean ± s.d. Source data provided as a source data file.

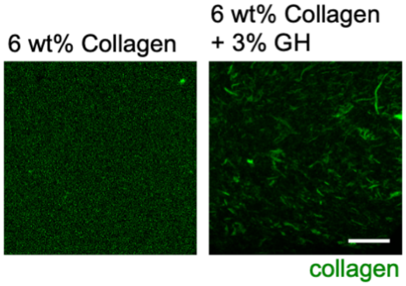

Supplementary Fig. 21: Collagen-based hydrogels do not form bicontinuous hydrogels. Representative single Z-sections of collagen hydrogels, with and without GH network. Scale bar = 50 µm.

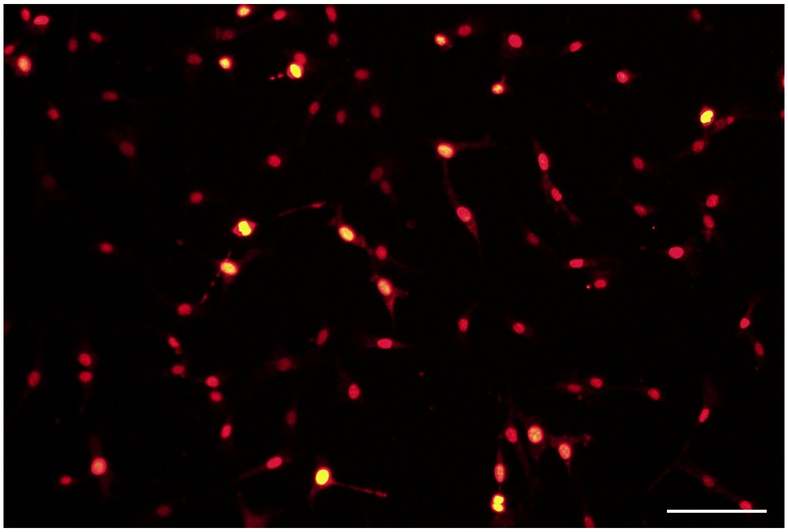

Supplementary Fig. 22: Validation of Ki67 antibody. Representative image of Ki67 (red). Scale Bar = 500 μm.
