# Supplementary material for: Microinterfaces in biopolymer-based bicontinuous hydrogels guide rapid 3D cell migration
Source: Nat Commun. 2024 Mar 29;15:2766. doi: 10.1038/s41467-024-46774-y (PMC10980809; doi:10.1038/s41467-024-46774-y)
Supplement: Supplementary file 3 — Description of additional supplementary files [file 41467_2024_46774_MOESM3_ESM.pdf]

DESCRIPTION OF ADDITIONAL SUPPLEMENTARY FILES

Title: Supplementary Movie 1| Bicontinuous hydrogel mixing protocol. Description: Droplet of gelatin and AD-HA solution (yellow droplet pipetted into syringe) is mixed with droplet of CD-HA and transglutaminase solution (clear droplet) with >10 circular motions to form a bicontinuous hydrogel.

Title: Supplementary Movie 2| Real-time fluorescent mixing of 0, 1 and 3% GH hydrogels. Description: Single Z-section imaged in real-time starting at 10 minutes after initial mixing (GR domains: green; GP domains: unlabeled). The first set of images correspond to 10-15 minutes after mixing. The second set of images correspond to 60-65 minutes after mixing. Each frame corresponds to 1 minute. Scale bar = 100 µm.

Title: Supplementary Movie 3| Visualization of Bicontinuous structure in 3D. Description: 3D reconstructions of confocal fluorescence stacks (spanning 100 µm) of 0, 1 and 3% GH hydrogels (GR domains: green; GP domains: grey). In 0% group, no GP regions exist. Scale bar = 50 µm.

Title: Supplementary Movie 4| Real-time MFC cell outgrowth and interaction with bicontinuous hydrogels. Description: Single Z-section of embedded MFC spheroids (GR domains: green; GP domains: unlabeled; actin: magenta) with cells infiltrating hydrogels from 8-75 hours. Each frame corresponds to 1 hour. Scale bar = 100 µm.

Title: Supplementary Movie 5| Visualization of Cell Outgrowth and Migration Tracks in 3D. Description: 3D reconstruction of confocal fluorescence stacks of real-time cell outgrowth (left) and corresponding visualization of individual cell tracks (multicolor lines) over a 72 hour culture period. Each frame corresponds to 1 hour. Scale bar = 100 µm.

Title: Supplementary Movie 6| Real-time cell migration with corresponding bead displacement. Description: Representative 5 μm maximum Z projection of a cell migrating through a 3% GH hydrogel (left, GR domain: green, actin: magenta) and corresponding particle image velocimetry (right, colored arrows with magnitude normalized per frame) over time. Each frame corresponds to 10 minutes. Scale bar = 50 μm.

Title: Supplementary Movie 7| Visualization of Gelatin-Agarose Particle Composite Hydrogels in 3D. Description: 3D reconstructions of confocal fluorescence stacks (1000 x 1000 x 100 µm) of low, medium, and high densification of composite hydrogels (gelatin: green; agarose particles: grey). Scale bar = 100 µm.
