# Supplementary material for: Microinterfaces in biopolymer-based bicontinuous hydrogels guide rapid 3D cell migration
Source: Nat Commun. 2024 Mar 29;15:2766. doi: 10.1038/s41467-024-46774-y (PMC10980809; doi:10.1038/s41467-024-46774-y)
Supplement: Supplementary file 11 — Reporting summary [file 41467_2024_46774_MOESM11_ESM.pdf]

nature portfolio

Corresponding author(s): Jason Burdick and Robert Mauck

Last updated by author(s): 02/05/24

Reporting Summary

Nature Portfolio wishes to improve the reproducibility of the work that we publish. This form provides structure for consistency and transparency in reporting. For further information on Nature Portfolio policies, see our Editorial Policies and the Editorial Policy Checklist.

Please do not complete any field with "not applicable" or n/a. Refer to the help text for what text to use if an item is not relevant to your study.
For final submission: please carefully check your responses for accuracy; you will not be able to make changes later.

Statistics

For all statistical analyses, confirm that the following items are present in the figure legend, table legend, main text, or Methods section.

n/a	Confirmed	
☐	☑	The exact sample size (*n*) for each experimental group/condition, given as a discrete number and unit of measurement
☐	☑	A statement on whether measurements were taken from distinct samples or whether the same sample was measured repeatedly
☐	☑	The statistical test(s) used AND whether they are one- or two-sided<br>*Only common tests should be described solely by name; describe more complex techniques in the Methods section.*
☑	☐	A description of all covariates tested
☑	☐	A description of any assumptions or corrections, such as tests of normality and adjustment for multiple comparisons
☐	☑	A full description of the statistical parameters including central tendency (e.g. means) or other basic estimates (e.g. regression coefficient) AND variation (e.g. standard deviation) or associated estimates of uncertainty (e.g. confidence intervals)
☐	☑	For null hypothesis testing, the test statistic (e.g. *F*, *t*, *r*) with confidence intervals, effect sizes, degrees of freedom and *P* value noted<br>*Give P values as exact values whenever suitable.*
☑	☐	For Bayesian analysis, information on the choice of priors and Markov chain Monte Carlo settings
☑	☐	For hierarchical and complex designs, identification of the appropriate level for tests and full reporting of outcomes
☑	☐	Estimates of effect sizes (e.g. Cohen's *d*, Pearson's *r*), indicating how they were calculated

Our web collection on statistics for biologists contains articles on many of the points above.

Software and code

Policy information about availability of computer code

Data collection	400 Bruker (NMR Acquisition), TA Instruments AR 2000 ex (Rheometer), TA Instruments DMA Q800 (Uniaxial Compression Testing), Bruker's Dimension Icon AFM (AFM nanoindentation), Leica TCS SP5, Nikon Ax 1 (Confocal microscopes), Zeiss Axioscan Z1 (Slide Scanner)
Data analysis	MATLAB_R2021B and ImageJ 1.53t (Image processing and analysis), Imaris 9.8 (Confocal reconstructions and volume analysis), GraphPad Prism 9.3.1 (statistical analysis), Adobe Illustrator 27.1.1 (Figure assembly and design), Topspin 4.1.4 and MestReNova 14.2.0-26256 (NMR software), Microsoft Excel 16.7.6 (data handling)

For manuscripts utilizing custom algorithms or software that are central to the research but not yet described in published literature, software must be made available to editors and reviewers. We strongly encourage code deposition in a community repository (e.g. GitHub). See the Nature Portfolio guidelines for submitting code & software for further information.

Data

Policy information about availability of data

All manuscripts must include a data availability statement. This statement should provide the following information, where applicable:

- Accession codes, unique identifiers, or web links for publicly available datasets
- A description of any restrictions on data availability
- For clinical datasets or third party data, please ensure that the statement adheres to our policy

All the data generated or analysed during this study are included within this article and its Supplementary Information. Additional information is available from the corresponding author on request.

Research involving human participants, their data, or biological material

Policy information about studies with human participants or human data. See also policy information about sex, gender (identity/presentation), and sexual orientation and race, ethnicity and racism.

Reporting on sex and gender	This study did not involve human participants.
Reporting on race, ethnicity, or other socially relevant groupings	N/A
Population characteristics	N/A
Recruitment	N/A
Ethics oversight	N/A

Note that full information on the approval of the study protocol must also be provided in the manuscript.

Field-specific reporting

Please select the one below that is the best fit for your research. If you are not sure, read the appropriate sections before making your selection.

☑ Life sciences ☐ Behavioural & social sciences ☐ Ecological, evolutionary & environmental sciences

For a reference copy of the document with all sections, see nature.com/documents/nr-reporting-summary-flat.pdf

Life sciences study design

All studies must disclose on these points even when the disclosure is negative.

Sample size	Sample sizes were not calculated a priori but were based on previous research from the lab (e.g. Taimoor Qazi et al. Advanced Materials 2021, Kwang Hoon Song et al. Advanced Healthcare Materials 2020).
Data exclusions	Structural data and spheroids were excluded from statistical analyses based on the robust regression and outlier removal test. These criteria were not established a priori. No animals were excluded. Explants from in vivo studies were excluded based on qualitiative visualization of missing hydrogel in defect space.
Replication	Replication and repeatability was confirmed through the use of multiple scaffolds or animals for each analysis, and experiments were repeated multiple times. Cells for scaffold-based assays were used from at least 2 donors for each analysis, and each analysis was repeated in full or in part through multiple studies.
Randomization	Hydrogels, spheroids, and animals were randomly assigned to treatment groups or conditions.
Blinding	Blinding was not used in this study. All analyses were quantitative in nature, based on either established techniques or on techniques described in the Supplementary Information. There were no subjective or qualitative analyses where decision-making by the researchers would have been required or could have impacted the findings.

Reporting for specific materials, systems and methods

We require information from authors about some types of materials, experimental systems and methods used in many studies. Here, indicate whether each material, system or method listed is relevant to your study. If you are not sure if a list item applies to your research, read the appropriate section before selecting a response.

Materials & experimental systems

n/a	Involved in the study
☐	☑ Antibodies
☑	☐ Eukaryotic cell lines
☑	☐ Palaeontology and archaeology
☐	☑ Animals and other organisms
☑	☐ Clinical data
☑	☐ Dual use research of concern
☑	☐ Plants

Methods

n/a	Involved in the study
☑	☐ ChIP-seq
☑	☐ Flow cytometry
☑	☐ MRI-based neuroimaging

Antibodies

Antibodies used	Ki67: Abcam, Ab15580, Lot #: 1015496-4, Dilution 1:500; CD-68:Biorad, MCA341GA clone ED1, Lot #: 159320, Dilution 1:500
Validation	Ki67 was validated in sFig. 22. CD68 antibody was validated as described in Qazi et al, Advanced Materials, 2021

Animals and other research organisms

Policy information about studies involving animals; ARRIVE guidelines recommended for reporting animal research, and Sex and Gender in Research

Laboratory animals	NIH nude rats, male, 9-12 weeks, were used in this study
Wild animals	The study did not involve wild animals.
Reporting on sex	All animals were male.
Field-collected samples	The study did not involve samples collected from the field.
Ethics oversight	The animal portion of this study was approved by the University of Pennsylvania Institutional Animal Care and Use Committee.

Note that full information on the approval of the study protocol must also be provided in the manuscript.

This checklist template is licensed under a Creative Commons Attribution 4.0 International License, which permits use, sharing, adaptation, distribution and reproduction in any medium or format, as long as you give appropriate credit to the original author(s) and the source, provide a link to the Creative Commons license, and indicate if changes were made. The images or other third party material in this article are included in the article's Creative Commons license, unless indicated otherwise in a credit line to the material. If material is not included in the article's Creative Commons license and your intended use is not permitted by statutory regulation or exceeds the permitted use, you will need to obtain permission directly from the copyright holder. To view a copy of this license, visit http://creativecommons.org/licenses/by/4.0/
